# Redesigning Rural Acute Stroke Care: A Person-Centered Approach

**DOI:** 10.3390/ijerph20021581

**Published:** 2023-01-15

**Authors:** Sarah J. Prior, Carey A. Mather, Steven J. Campbell

**Affiliations:** 1 Tasmanian School of Medicine, Rural Clinical School, University of Tasmania, Burnie, TS 7320, Australia; 2 Australian Institute of Health Service Management, University of Tasmania, Launceston, TS 7250, Australia; 3 School of Nursing, University of Tasmania, Launceston, TS 7250, Australia

**Keywords:** health service improvement, healthcare staff, qualitative, patient journey, person-centered, redesign, rural, stroke

## Abstract

Stroke service delivery in rural areas in Australia lacks evidence-based, best practice care protocols as a result of limited resources and opportunity. Healthcare redesign is an approach to improving health services by understanding barriers and enablers to service provision and work with users to develop solutions for improvement. This research aimed to qualitatively evaluate stroke care in rural Tasmania using a person-centered approach, as part of a larger healthcare redesign initiative to improve acute stroke care. Semi-structured interviews, aimed at gaining insight into experiences of healthcare staff and users, were conducted. Thematic analysis revealed three global themes (communication, holistic care, and resourcing) that demonstrated some consistency between healthcare staff and user experience, highlighting that some needs and expectations were not being met. Results of this experiential study provide important perspectives for delivering needs-based improvements in service provision for acute stroke care. Overall, this study showed that systems of stroke care in rural areas could be improved by utilizing a redesign approach including healthcare staff and users in the development of solutions for health service improvement.

## 1. Introduction

Stroke is one of Australia’s leading causes of death and disability [1]. Evidence suggests that care in a structured, multidisciplinary, dedicated stroke unit improves survival and independence rates by up to 20% [2] although lack of access to stroke units, particularly in rural areas remains an issue. Rural Australia has noticeable disparities in healthcare with higher numbers of stroke hospitalizations than metropolitan Australia [3]. Rural and remote Australians live shorter lives, and they have increased levels of chronic disease, poorer mental health, and reduced access to health services compared to metropolitan populations [4]. Rural and remote stroke patients often have poorer health outcomes as they are less likely to receive timely evaluation, assessment, or treatments such as intravenous thrombolysis or endovascular therapy [5]. Limited resources, an aging population, and ongoing difficulties in recruiting and retaining healthcare staff potentially contributes to these disparities as well as a poor user experience. The Institute of Medicine [6] describes patient and family-centered care as “a partnership among practitioners, patients, and their families to ensure that decisions respect patients’ wants, needs, and preferences, and that patients have the education and support they need to make their own decisions and participate in their own care” (p15). The term “user” [7] represents patients, families, and carers in this context, as co-design encourages person-centered, participatory collaboration among stakeholders [8]. Including users in research or other quality improvement initiatives creates opportunity for health services to make improvements that align with the needs and expectations of these stakeholders [8].

Healthcare redesign is a process that aims to improve health services through identifying issues and root causes and designing evidence-based solutions. This process includes scrutinizing care quality, resourcing, utilization of current resources, and quality assurance from the perspective of users and organizations. Utilizing a person-centered approach by exploring the patient journey is a means to ensuring meaningful and sustainable solutions to issues affecting user experience and contributing to healthcare staff dissatisfaction. A person-centered approach can be defined as ensuring that individuals are treated respectfully with an understanding of “what is important to the patient, their families, carers, and support people” and are the focus of this study [9]. Benefits of involving users in healthcare redesign have been highlighted previously [10], including improved sustainability of quality improvement implementation, better quality care and increased quality health, and user information addressing specific concerns. The patient journey approach, developed in the United Kingdom (UK), has been integral in the successful redesign of a number of healthcare services, including acute stroke care, in a large city hospital service [11]. Furthermore, redesign initiatives [12,13,14] suggest improved access to health services and improved health and quality of life of users are prominent outcomes in the patient journey approach. This method also places emphasis upon the involvement of healthcare staff, i.e., any staff working in a health service that is involved in the care of users. Whilst there is much research around the involvement of users in redesigning healthcare services, there is little to show the value of direct staff input. The engagement of clinicians and senior administration is often described as playing an important role in healthcare redesign with point-of-care nursing and allied health staff often overlooked. Staff members are the key stakeholders in healthcare redesign as there is an increased need for change at organizational and departmental levels, and their involvement and their views are more likely to lead to active engagement and improved sustainability in change and user outcomes. The clinical viewpoint is a valuable tool for developing services that are functional and evidence-based, as well as accepted by local staff teams.

The aim of this study was to utilize a person-centered approach to explore and understand experiences of healthcare staff caring for acute stroke patients, and users of acute stroke care in rural Tasmania, Australia.

## 2. Materials and Methods

### 2.1. Study Design

This qualitative study utilized semi-structured interviews to capture and explore experiences of stroke patients, their families, and carers (hereafter known as users) and healthcare staff working with stroke in a rural area. This qualitative study was nested in a larger, mixed-methods study that utilized a broad range of organizational and health outcome quantitative data. The larger mixed-methods, healthcare redesign study aimed to improve acute stroke services by developing a clear understanding of the needs of the stroke service users and healthcare providers pertaining to acute stroke care and acute stroke services in this area. This project was divided into two main parts:

1. A qualitative research component consisting of patient and family interviews, as well as healthcare staff interviews. The aim of this part of the project was to report on the in-hospital experiences of those receiving and delivering stroke care in Northwest Tasmania.
2. A quantitative component highlighting the current state of acute stroke services in Northwest Tasmania, as well as providing a baseline for comparative analyses following the implementation of a redesigned acute stroke care service.

### 2.2. Setting

Tasmania, Australia’s island state, has lower general health and fewer medical practitioners per capita excluding Western Australia, than other states in Australia, with the residents in rural areas having the worst health [15]. Tasmania has 23 public hospitals with four major public hospitals located in or around the major cities. At the time of this study, only two hospitals were currently listed as having formal stroke units.

The study area is classified as a rural area, covers a population of approximately 114,000 people, and includes two major public hospitals. These hospitals combined admitted, on average, more than 180 patients diagnosed with stroke during the financial year (2016–2017), and, at the time of this study, neither had a dedicated stroke unit or multidisciplinary stroke team available, despite the recommendations by the Australian Stroke Foundation [16] that hospitals with more than 75 stroke presentations per year should have a dedicated primary stroke care capability.

### 2.3. Stakeholder Group (Patient Journey Group)

The patient journey group, consisting of senior medical, nursing, allied health, and executive staff, as well as the research team, met monthly to discuss sampling, progression of the project, and the outcomes of the interviews. This group comprised a range of stakeholders (hereafter known as staff) who had involvement in the acute stroke patient journey, including administration, clinical and nonclinical roles, and executive roles.

### 2.4. Participants

#### 2.4.1. Staff

Twenty-seven health professionals from hospitals and general practices/community care were invited to participate in an interview. Staff member roles identified in this group defined above were known to work with acute stroke patients across the region.

#### 2.4.2. Users

One hundred users who had utilized stroke services in the study area within the previous 6 months were identified by diagnosis-related groups (DRGs) in the health service digital medical records system.

### 2.5. Recruitment

#### 2.5.1. Staff

Staff members from both major hospitals in the region, as well as general practitioners from community practices, were invited to volunteer as participants to explore the current systems of stroke care. An email invitation, including an information sheet and consent form, was emailed to all staff who had worked in stroke care as guided by the stakeholder group. Interested staff contacted the research team and arranged a time to meet. During each interview, staff members were asked to provide their feedback on the current services, as well as their thoughts about user involvement, and they were given an opportunity to put forward ideas about a proposed dedicated stroke service in the region.

#### 2.5.2. Users

The matrix sampling method [17], a form of stakeholder sampling, was utilized to select users for interview. This purposive sampling method allowed the project team and stakeholder group, to determine the parameters around which users should be interviewed. This method also determined a relevant sample size for the population on the basis of the study characteristics. The following recruitment strategy was undertaken:

1. An information sheet was mailed to these users. This sheet informed and invited users to provide consent for further data collection regarding their health history pertaining to stroke only. Consent was provided in writing.
2. Participation selection, from those consented, was according to the criteria identified in the sampling matrix (Table 1). These details were identified from an audit of the digital health records.

Eligible users were contacted by telephone to arrange in-home interviews. All eligible participants were required provide further written consent prior to participation in the interview process. Any users who did not fit the specified matrix criteria or were surplus to needs based on the sampling matrix were notified by mail.

### 2.6. Method—Interviews

#### 2.6.1. Staff

Staff interviews were held during usual working hours, where possible, and in meeting rooms that were available. Interviews were recorded one on one, face to face, on a digital voice recording device. Interviews were semi-structured; the interviewer had a list of questions for which the answers were required but did not necessarily have to ask each question sequentially. Staff were asked to tell their story about stroke care, and any gaps were filled by asking additional questions.

#### 2.6.2. Users

Interviews were held in a place of choosing by the users. In most cases (22/27), users preferred to be in their homes; three users were interviewed via telephone (interstate residents), and, in the remaining two cases, users wanted to meet in a public café. Interviewees were asked to talk about their in-hospital experience with stroke care. Research ethics was approved from the Tasmania Health and Medical Human Ethics Research Committee (H0015964).

### 2.7. Data Analysis

Data adequacy was achieved through the purposive sample utilized for this qualitative study, despite all user fields not being met.

Analysis of interview data was performed in two parts. The first part of the thematic analysis for this project was based on an analytic tool developed in 2001 [18]. This method was based upon developing thematic networks and involved three main steps:

1. Breaking down the text—developing thematic network,
2. Exploring the text—describing thematic network,
3. Integration—interpreting the patterns within the thematic network.

A system of coding was developed, thereby allowing data to be divided into segments and allocated accordingly [18]. Refining and arranging these segments into themes gave the preliminary structure for overall themes. These themes were refined further into organizing themes, and global themes were deduced. Global themes brought together foundation and organizing themes into one or two overarching categories. Interpretation of global themes developed from this research comprises the results (Supplementary Materials Tables S1 and S2).

## 3. Results

### 3.1. Demographic Information

A total of 117 information sheets and consent forms were mailed to users who had been admitted to one of the local hospitals with a stroke during 2015 and 2016. Recruitment excluded any users who had died in hospital. Eight (6%) consent forms were returned to sender, one (0.8%) did not wish to participate, and 24 (21%) contacts were made by family members indicating that the user had died. A total of 32 (27%) users returned consent forms for participation in interviews; however, five were unable to continue through to the interview phase. Overall, a 23% rate of response was recorded. Digital medical records of all consenting participants were assessed, and eligibility for interview was determined by the matrix sampling method (Table 2). The average length of interviews was 38 min and 38 s. The average length of staff interviews was 24 min and 34 s.

Twenty-seven staff (100%) participated in an interview. Staff members were from across a range of disciplines working in stroke care (Table 2) on a regular basis, to ensure participation of stakeholders. The length of time that staff members were involved in stroke care ranged from 7 months to more than 20 years. Table 1 shows the range of staff who were interviewed and the healthcare organization that they were associated with. Representation was achieved from all health services and from a broad range of disciplines, relative to the number of staff within the rural health service.

### 3.2. Thematic Data

Table 3 shows organizing themes from staff interview data and user interview data side by side. Data show similarities in some aspects of current stroke care, highlighting some of the issues shared by both staff and users and showing some of the differences between the issues perceived. The global, overarching themes are presented (Table 3).

### 3.3. Global Themes

#### 3.3.1. Communication

Inconsistent communication was discussed in both cohorts with a strategic focus from staff and a more subjective and personal range of issues from users. Lack of effective early communication strategies was identified as a weakness from staff, across disciplines. Similarly, having limited standardized decision-making tools created consistent communication challenges. Users highlighted that information was a key component of their early recovery, and they relied on a consistent communication to help them understand their current state and expectations around physical, emotional, and mental recovery, moving forward. Users described that feeling time was a factor that influenced communications they received from healthcare providers, and that fragmented and delayed communication often resulted in fear, stress, and impatience.

*“Stroke care is not bad, but we have our hands tied”* Staff Interview #9.

*“There is no personal communication between the hospital and GPs”* Staff interview #13.

*“Communication among the patient, family, and staff is a priority”* User Interview #17.

*“The nurse didn’t explain why she was giving me medication—she got upset when I questioned her about it”* User interview #2.

*“Communication with the family was dreadful”* User interview #10.

*“I didn’t get any information about strokes. I just wanted to know what caused it, why it happened, but I didn’t get any answers”* User interview #11.

Users often expressed fear when discussing their first interactions with stroke care. In many cases, users did not know what to expect, and no information was given to them. Users identified a lack of confidence and clinical acute stroke expertise of staff, which resulted in fragmented and delayed communication. This delay contributed to and continued to cause stress and angst among patients and families.

#### 3.3.2. Holistic Care

“Mental health matters” was a theme evident in both cohorts, and much discussion was devoted to this topic throughout many of the user and staff interviews. Discussions were similar; however, staff reported more service-related issues, whereas users focused on internal issues. Users identified that early mental health support was important in stroke recovery as often it is a life-changing event. Managing relationships and priorities internally can be challenging post stroke, and more emphasis on user mental health was a factor in post-stroke frustration and stress.

*“Get them in, get them well, get them out”* Staff interview #26.

*“A neuropsychologist would be beneficial for designing effective patient therapy programs”* Staff interview #25.

*“It’s quite a fight to keep going every day. Mental healthcare is lacking”* User Interview #5.

Staff discussions focused on alleviating stress and increasing motivation through information provision, education, and the use of planning strategies following the acute period. Users placed greater emphasis on activities of daily living, familial relationships, and support through follow-up conversations. Many users commented on the potential benefit of a telephone call, follow-up appointment, or a group session just as a check-in at the completion of their acute stay in a health service. Although users sometimes felt overwhelmed immediately after their stroke, they often had feelings of not wanting to bother anyone at the hospital with questions or queries about what was happening to them. Some users explained that this behavior led to feeling unsupported and somewhat depressed. In some cases, after the acute care had been completed, general practitioners were not aware of the stroke history of users nor offered any further support for users post stroke, which lead to users feeling alone.

Users in rural Tasmania indicated a more person-centered approach to care through recognizing and discussing human interactions and user follow-up, as well as person-centered information provision. However, many staff did recognize the need for following up with users post stroke.

*“Patient follow-up should be mandatory—especially for life-changing conditions”* Staff interview #19.

Education as a tool for receiving and providing right care at the right time was discussed in staff and user interviews. Staff felt that having more education, specifically around organized, multidisciplinary stroke care, would improve user expectations and outcomes. Staff placed great emphasis on acute stroke education, for themselves, their colleagues, and users. Similarly, users indicated that information provision could be improved, more so on the timing of and delivery of content, again highlighting that users want a more person-centered approach rather than clinical care delivery. Staff indicated that, in many cases, there was a lack of confidence speaking with patients about stroke as they did not feel clinically educated to an adequate level to provide this information.

#### 3.3.3. Resourcing and Service

Research indicated that staff members were under pressure regarding the delivery of acute stroke care. Limited resources, experience, and poor support for rural healthcare settings were identified as major contributors. However, local users were supportive of their hospitals and understanding of the pressures that staff members were under to provide high-quality care despite service gaps. Although staff spoke of acute service inefficiencies more than patients, the need for reliable and continuous service provision was more prominent in the user interview data.

Overall service expectations centered around previous experience, and there were clear distinctions between staff and user responses. A service-focused, strategic, and educational view of holistic healthcare was evident in data collected from staff interviews, particularly around ongoing quality care eluding to organized stroke care saving lives.

*“There is no protocol, no stroke pathway, and no thrombolysis program”* Staff interview #10

*“There is no orientation for nurses around stroke care and no specific training; staff are lacking in confidence”* Staff Interview #2

*“Good planning equals good implementation”* Staff interview #17

*“The ladies came around but did nothing”* User interview #4

As a global theme, acute stroke service provision and reliability of stroke services were the focus of many discussions around resourcing, unavailability, and continuity of care. Staff indicated that resources available to them are inadequate for providing best practice stroke care [16]. Often, they felt disappointed that they were unable to better help users. Users’ perceptions of acute stroke service provision and reliability of stroke services were dependent on previous experiences in healthcare overall, not just stroke care. Some users suggested that services they received, including daily visits from medical practitioners, nurses, and allied health staff, were above and beyond expectations. They indicated that relationships built with these staff members helped to keep them motivated and continue with their rehabilitative care. Other users commented that staff members were doing the best they could, with the little resources they had available. It was noted that there was some frustration around periods of service unavailability (weekends and public holidays) as users lost control of their routines, which is particularly important in stroke care.

There were, as expected, many positive comments from users about care received during and following stroke. These comments often reflected the performance of individuals, rather than the system as a whole.

*“The doctor came around and we had a long chat which I appreciated. It was the most valuable chat I’ve had with anyone”* User interview #15

“Care in ** Hospital was good—nothing to improve” User interview #11

*“I was treated respectfully, and my privacy was protected”* User interview #13

## 4. Discussions

The aim of this study was to explore and understand user and healthcare staff experiences of acute stroke care for the purposes of contributing to the redesign of current stroke services in rural Tasmania. Engagement at many levels is crucial for successful redesign, implementation, and sustainability of healthcare services. Results from this study suggest that healthcare staff and users in rural Tasmania share many similar views on challenges in stroke service delivery, consistent with findings from a previous study [19]. These results reflect acute service delivery issues present in several rural health service areas across Australia [20] and highlight the importance of understanding the needs of users, including healthcare staff, in acute care. This information is often missing from health service improvement projects but is valuable to showcase the needs and expectations of users [21] and staff working with users. Whilst qualitative data can be further supported by quantitative data, this study aimed to demonstrate user experience, and to understand barriers and enablers to delivery of safe, appropriate, and high-quality care.

Communication among staff and between staff and users was shown to be an issue in acute stroke. Interviews with staff suggested that there were few viable strategies in place for ensuring effective communication between and among staff, with mention of hierarchical decision making and lack of consultation around best practice care for users. Consistent with previous work [19], recommendations for improved communications and multidisciplinary care were put forward. It was suggested that a lack of time and frequent staff shortages contributed to minimal communication among staff and between staff and users. These issues are global with the time constraints and low staff numbers anecdotally reported in many healthcare facilities. Morris et al. [19] found that a lack of information about stroke and treatment in an acute setting engendered anxiety: “I don’t think in the hospital they work as a team they didn’t pass any information to each other”. Described as the overlooked rehabilitation tool [22], communication is an important part of any healthcare experience, particularly in life-changing conditions such as stroke. Consistent with findings, users in other studies felt that it was the role of medical practitioners and hospitals to provide information, explanations, encouragement, and advice, with almost half believing that this need was not being met [23,24,25]. A further study [26] suggested that “despite widespread evidence of the need to improve information-giving for stroke patients and carers, relatively few evaluations of the content and methods of delivery have been undertaken” (page 129). Specific, high-quality information can allow patients to take more responsibility for their health, respond actively to arising health issues [27], feel like they have more control [28], create positive attitudes toward their illness, and participate effectively in decision-making processes [29].

Experiential interview data found that staff and users have differing areas of priority in relation to stroke service delivery. Users place a great emphasis on the personal aspects of stroke care including kindness and the conversations staff have with them, as well as the in-hospital support provided. This behavior is often typical of rural and remote areas where comfort is often found in building relationships and being informed by people who are known to you, or who you have had previous healthcare experiences with [30]. In contrast, clinical staff identified that time spent with patients should be utilized to provide education and to focus on clinical tasks and being able to offer professional services for mental wellbeing. As a result of clinical training and wanting to provide the best care for patients, staff members believed that this is the best way to deliver stroke care. However, this also highlights that the patient is not involved at a high enough level of consultation and decision making for staff to really understand user needs. Person-centered care principles would really benefit staff working with individuals to further understand their values, expectations, and preferences for treatment. Moore et al. [31] suggested that users of healthcare such as patients, families, and caregivers are largely ignored in the implementation of healthcare practices, and that active engagement of these stakeholders should be encouraged to improve patient safety and quality of care. Qualitative research has highlighted the importance users place on building relationships with their clinical teams and personal care over clinical care—information that is not clearly articulated in quantitative data.

Current opportunities for stroke specific clinical education in rural Tasmania are limited due to course delivery and time constraints, as well as location. However, it was noted throughout this study that the National Stroke Foundation (Australia) offers a range of online stroke specific education modules suitable for all healthcare staff. Similar findings were reported in a previous stroke services study [32], suggesting that nurses found that a lack of training, time, and skills were perceived as barriers to providing effective care for depressed users. Improving opportunities for staff education could improve education for users through giving staff more confidence to have a conversation with users and provide an opportunity to ask questions, develop plans, and discuss their future. Nursing education and better staff training were also identified as recommendations for change during staff interviews in a review by Java et al. [33]. An identified lack of information provided to users in this study was attributed to limited knowledge and skills; therefore, a suggestion to establish a dedicated stroke service could potentially alleviate these problems. The staff group in this study also expressed the value of a specialist stroke pathway and opportunities to develop specialist knowledge, skills, and resources.

Limited accessibility to mental health services was a major topic of discussion across both staff and user interviews. Despite slightly different approaches to this topic, overall, provision of suitable mental health support and follow-up post stroke was deemed to be absent. In rural health areas, where services are often under-resourced, this type of practice may not be feasible; however, a plan for mental health support should be in place as part of the ongoing rehabilitative processes as per national guidelines [16]. Post-stroke depression occurs in up to one-third of all ischemic stroke survivors [34] and has been linked to worse functional outcomes, slower recovery, and decreased quality of life [35,36]. In this study, patients differed in their approaches to stroke recovery, with some users being very involved in the acute phase, motivated and working hard to improve long-term outcomes, whereas some users felt as though everything should be taken care of by others. Reasons for these differences were not explored in this study, but previous health service experiences and generational influence may relate to the expectations of users. Victoor’s [30] patient choice of healthcare provider study suggested that patients who have previously been treated in a health service with a specific type of care (patient-centered or authoritative) expect each subsequent experience to be similar. It was expected that interviewing users would show they would like to be more actively involved in care whilst in hospital, even if this meant changing the way in which they interacted with previous healthcare providers. However, the results suggested that most stroke patients interviewed preferred to have clinical teams make decisions for them, as they felt they knew best. Users also preferred to leave their care regime to clinical teams and not to have to worry about organizing their own care. These factors may be due to the specific demographic of the population of stroke patients in rural Tasmania. These patients were generally older, did not spent a lot of time in hospitals, and had low health literacy when compared to other Australian populations [37]. Findings are contradictory to other research suggesting that involving users in their own care, particularly decision making, is an important part of the rehabilitation and recovery process from health issues [38]. Whilst not excluding person-centered practices such as reaching a shared understanding with users and developing goal directed approaches for user recovery, this study showed that users were more likely to feel comfortable with services if they felt like they were able to make the best decisions on their behalf. Perhaps a generational view or that of a population that does not feel equipped to be so involved in their clinical care highlights the need for further user engagement research in healthcare in this and other rural health service areas with similar populations.

Several barriers and enablers to stroke service provision in regional Tasmania from a number of perspectives were highlighted. However, these results likely reflect those of many similar sized rural health districts, at least within Australia and potentially on an international level. A 2011 review [39] suggested that there were several means to narrowing gaps in health outcomes for stroke between metropolitan and rural health service areas including government policies and funding, clinician-driven initiatives, adopting evidence-based guidelines and management protocols, and ensuring ongoing clinician education.

### 4.1. Limitations

Matrix sampling may be a limitation, as it may not be possible to recruit participants to each selected criterion, leaving gaps in data. In this study, it was noted that the “patient transfer” criteria were more difficult to recruit to due to smaller numbers of admitted patients fitting this description within the sample population. Utilizing the same method in the UK, Campbell et al. (2004) also encountered difficulties in recruiting participants to fulfill all criteria. Despite not being able to include users from these criteria, the stakeholder group agreed that the sample reflected a representative population, which was the key purpose of the matrix sampling approach.

This study was undertaken prior to the COVID-19 pandemic, and circumstances regarding changes to models of care may have occurred, reducing generalizability. Recent research indicates that chronic disease management has been further impacted by resourcing due to the COVID-19 pandemic and the sequalae associated with the rise of long COVID-19, which has probably exacerbated these findings, rather than diminished their timeliness [40]. Additionally, this regional area was the site of the first nosocomial outbreak of COVID-19, which has potentially deepened impact of lack of resources in this area [41]. This study strengthens current evidence for ongoing digital support in acute care to improve accessibility in rural areas, as well as more broadly.

### 4.2. Future Directions

Results from this study, and the larger mixed-methods redesign initiative has enabled the Tasmanian health service (Northwest) to develop evidence-based solutions and make significant changes to how acute stroke care is delivered. Although a formal stroke unit is not yet established, users in this area now have access to a 24 h neurology service via a telehealth model and a 24 h state-wide neuroradiology service. These improvements are reducing the long-term costs required to care for users with untreated strokes and ensure that safe, high-quality care is delivered to those diagnosed with an acute stroke.

## 5. Conclusions

Rural health services in Australia face many challenges in delivering acute stroke care. Users and healthcare staff in rural Tasmania have highlighted barriers and enablers in accessing and delivering relevant, person-centered care that is timely and evidence-based. Improvement of healthcare services incorporating views of healthcare staff and users through the redesign approach creates opportunities to better understand local needs and expectations, as well as build these into improved or new models of care.

## Figures and Tables

**Table 1:** Sampling matrix.

	≤1 Medical Risk Factor and/or ≤1 Lifestyle Risk Factor		>1 Medical Risk Factor and/or >1 Lifestyle Risk Factor	
Medical risk factors	TIA, atrial fibrillation, diabetes, fibromuscular dysplasia			
Lifestyle risk factors	High blood pressure, high cholesterol, smoking, excessive alcohol consumption, obesity, lack of exercise			
	**65+ years**	**<65 years**	**65+ years**	**<65 years**
Ischemic	3	3	3	3
Hemorrhagic	1	1	1	1
Transferred From H1/H2 **	1	1	1	1
Transferred to H1/H2 ***	1	1	1	1
Bought in by ambulance (BIBA)	1	1	1	1
Private car	1	1	1	1
Mild severity *	1	1	1	1
Moderate severity *	1	1	1	1
Severe *	1	1	1	1

* Measured via functional independence measure (FIM) score; ** transferred to a larger tertiary hospital; *** transferred from a smaller rural health service.

**Table 2:** Staff participation list for interviews.

Staff Role	Number of Interviews	Healthcare Organization
Registered nurse	9	H1/H2
Enrolled nurse	1	H1
Consultant (general medical)	1	H2
Registrar	1	H1
Physiotherapist	2	H1/H2
Occupational therapist	2	H1/H3
Speech pathologist	2	H1/H2/H3
Social worker	2	H1
Clinical nurse educator/coordinator	2	H1/H2
General practitioner	2	H3
Pharmacy	2	H1
Radiology	1	H3

H1 = hospital 1, H2 = hospital 2, H3 = all healthcare staff outside hospital environment.

**Table 3:** Organizing themes from interview data.

Staff	Users
Health education creates a healthy environment	Perception of overall healthcare depends on experience
Organized stroke care saves lives	Information is a key component of recovery
Communication is a strength	Channels of communication influence experience
Mental health matters	Mental health matters
Service inefficiencies	Reliability of service provision creates a motivational arena for users
Ongoing, quality care	User follow-ups provide a sense of support and reassurance
	Human interactions are an integral part of a healthcare service

## Data Availability

The aggregated data in this study are available as supplementary files. Raw data are not publicly available as per our ethics approval and privacy restrictions.

## Supplementary Materials

The following supporting information can be downloaded at https://www.mdpi.com/article/10.3390/ijerph20021581/s1: Table S1. Thematic analysis for user interview data; Table S2. Thematic analysis for staff interview data.
