# Supplementary material for: Redesigning Rural Acute Stroke Care: A Person-Centered Approach
Source: Int J Environ Res Public Health. 2023 Jan 15;20(2):1581. doi: 10.3390/ijerph20021581 (PMC9864304; doi:10.3390/ijerph20021581)
Supplement: Supplementary file 1 [file ijerph-20-01581-s001.zip › ijerph-2093591-supplementary.pdf]

Supplementary File Table S1: Thematic Analysis for User Interview Data

Themes	Organising Themes
1. Patient experience in the healthcare system is dependent on the level of health literacy, previous experiences and age<br>2. Individually, the staff are often commended for their work<br>3. As a business, the healthcare system should be allocated more money, services and resources<br>4. Perception of service availability and suitability differs between patients	Views of healthcare are dependent on knowledge and age<br>Healthcare needs more resources<br>Perception of healthcare depends on experience
1. Early rehabilitation = better patient outcomes<br>2. Patients are often concerned when staff can't (or won't) tell them what is happening<br>3. Patients do not like to ask for help or assistance with thing that they think they should be able to do on their own<br>4. Patients are often in shock, surprised when they find out they have had a stroke.<br>5. Patients are often unsure of what to expect following their diagnosis<br>6. Patients are often frightened by the word stroke<br>7. Early diagnosis can be reassuring<br>8. Lots of tests can make the patient feel as though something is being done to help them<br>9. Being in a local hospital with familiar faces and close to family helps patients to feel less stressed and less frightened<br>10. Patients feel that starting physio/other services early in the admission is beneficial and makes them believe that they can't recover.<br>11. The meals were fantastic and help with patient outcomes<br>12. Human kindness plays a major role in care	Patient outcomes are improved with early treatment and rehabilitation<br>Patients rely on a steady stream of information and service delivery to help them understand expectations and recover<br>Being close to home, with familiar faces and places is important to patients<br>Kindness improves experience<br>Information is a key component of recovery<br>Human interactions are an integral part of health care
1. Patients are often given too little information directly from doctors and nurses<br>2. Some patients and their families appreciate the Stroke Pack, whereas other find it frightening, too hard to read or not useful.	Lack of specialised clinical expertise creates a stressful environment for patients and their families<br>Time constraints means that patients are not as well informed as they feel they should be.

3. Patients often find that staff are not able to give them specific information about their condition due to a lack of clinical knowledge<br>4. Patients feel that there is not enough time to have discussions with staff regarding their condition<br>5. The staff turnover means that new staff are learning about a patient every time they come in<br>6. Families often feel frightened and confused due to a lack of explanation and one on one education<br>7. Patients without family/advocacy feel scared and confused by the lack of direct information<br>8. Communication between doctors and nurses is often fragmented<br>9. Communication with GP following discharge is hit and miss<br>10. Having social interactions with other patients can positively contribute to the information about stroke recovery and associated problems.<br>11. Patients feel that the doctors often talk to their students rather than to them.<br>12. The time it takes between staff discussing a diagnosis is often long and creates a stressful environment for patients and their families.<br>13. Education of family and carers is an important part of rehabilitation and creates improved patient outcomes<br>14. Discharging a patient (to a nursing home) without family knowledge creates a stressful situation	Fear is associated with a lack of information provision in hospital and following discharge.<br>Fragmented and delayed communication causes stress and impatience.<br>Channels of communication influence patient experience
1. Patients feel that the hospital environment in the acute setting is very busy and that they do not want to interrupt staff<br>2. Patients rate the services available depending on their knowledge outside of one hospital. Many patients consider the services exceptional whilst others felt they are severely lacking in areas.<br>3. Some patients prefer a private room, sharing inly meals and social times with other patients while some patients prefer a shared room for the social contact.	Perception of service delivery is dependent on the experience of the patient with healthcare<br>Daily visits by allied health services create a positive experience for rehabilitation<br>Patients appreciate a service where they can build relationships with staff and other patients

4. Patients feel that staff are encouraging and help to motivate them to get moving again<br>5. Patients appreciate the reliability of allied health services (physio/OT) each day to aid in their rehabilitation<br>6. Patients feel that the services offered in H1 are better than those in other (bigger) hospitals, mainly due to a more personal experience.<br>7. A misdiagnosis can cause distress for patients and their families<br>8. A lack of weekend services discourages patients<br>9. Having to wait long periods for some assessments (swallowing) caused distress to patients as they were hungry/thirsty<br>10. Allowing the primary carer to visit and stay anytime (not just visiting hours) is a great benefit to the patient and to the family.	Periods of service unavailability can be stressful for some patients (weekends, after hours)<br>Reliability of service provision creates a motivational arena for patients
1. Patients often feel that a follow-up phone call from the hospital would be beneficial, particularly if they had questions<br>2. Families and carer's have indicated that a follow-up from the hospital or doctor would give them a chance to express any concerns or ask questions<br>3. Patients and their families often feel a lack of support when leaving hospital<br>4. Not all patients are interested or are able to access online support groups or chat lines as suggested in the Stroke Pack<br>5. Patients are often frustrated at the lack of information given to them on discharge about services available to them<br>6. Patients have found that there are many financial adjustments following a stroke, particularly around employment.<br>7. Planning for the immediate future after leaving hospital is lacking<br>8. Patients indicate that follow-up appointments or referrals are often not explained to them<br>9. GP's often have no information about their patients having a stroke or any medication changes	Follow up post-stroke should be a mandatory process<br>A lack of information provision and planning during or prior to discharge can be frustrating for patients and their families<br>Communication with GPs is lacking causing issues for patients<br>Patient and family follow ups provide a sense of support and reassurance

1. Patients do not always feel they have mental health support during their stay in hospital and following discharge<br>2. Patients often feel unable to ask about mental health services<br>3. Patients often feel depressed but do not want to bother anyone with their problems<br>4. Patients often feel lucky as there are always people worse off than they are, even though they may have lost a lot and feel sad about it<br>5. Patients often feel like they have lost their dignity and become frustrated by this<br>6. Patients indicated that they would like someone to talk to about their experience<br>7. Patients indicated that losing their freedom through deficits and disability was a life changing event that they could not always discuss with their family/carer.<br>8. Rebuilding relationships is a difficult time post-stroke<br>9. Home role reversal post-stroke can be very stressful for families and patients<br>10. Group therapy provides an avenue for rehabilitation outside of the home and the hospital	Mental health support is important for patients in stroke recovery<br>Patients often put others before themselves<br>Relationships can be difficult post-stroke<br>Stroke is a life changing event<br>Mental health matters

Supplementary File Table S2: Thematic Analysis for Staff Interview Data

**Basic Themes**	**Organising Themes**
1. Staff and patient education leads to better patient outcomes<br>2. Up to date stroke related resources create better patient experience<br>3. Educated staff = educated patients<br>4. Staff training specific to stroke leads to better patient outcomes and expectations<br>5. Patient and Family feedback provides opportunities for staff to improve skills and service delivery<br>6. Strengthening links with the Stroke Foundation would provide another resource avenue	Health education creates a healthy environment<br>Education as a health service tool<br>Education creates better patient outcomes

7. Ensuring doctors have sufficient time to spend with stroke patients to discuss their health, wellbeing and future would create a better rapport between doctor and patient/family.	
1. Coordinated care = better patient outcomes<br>2. Stroke service provision requires specialist functionalities<br>3. Quality management and leadership in stroke care creates better working environment<br>4. Collaboration with larger centres will allow staff to contact someone for information or for service referral if needed<br>5. Patients are waiting too long for services<br>6. Stroke patients require stroke nursing, not just ticking off of boxes on a checklist<br>7. Utilising technology in acute and rehabilitation services can improve patient outcomes<br>8. Utilising technology for communication can improve the relationships between the hospital and the community (GP's)	Organised stroke care saves lives<br>Organised multidisciplinary stroke care<br>Collaborative care<br>Technological advances
1. Effective communication ensures clearer decision making<br>2. Creating a social support system is beneficial for patient outcomes<br>3. Facilitation of stroke awareness events will involve community in stroke education<br>4. Management making decisions in consultation with staff, rather than for staff, will create a better working environment<br>5. A stroke coordinator making a phone call to a GP regarding the discharge of a stroke patient will ensure that the GP has up to date information and is able to provide optimum care when required.	Communication is a strength<br>Effective communication strategies<br>Improved decision making methods
1. Patients stays are prolonged due to delays in service provision<br>2. Inadequate funding for stroke care means inadequate service provision<br>3. Ensuring that patient programs (e.g. hydrotherapy) continue, enables NW patients to access services close to home within a good timeframe.	Service inefficiencies<br>Service provision is dependent upon resources

1. Isolation and depression are common in stroke patients<br>2. Letting patients do things for themselves, albeit a slow process, provides optimum rehabilitative services<br>3. Inviting patients and families/carers to "huddles" or ward meetings will allow information to be disseminated effectively and create a more holistic approach to stroke care<br>4. Utilising patient interests in rehabilitation activities can assist in keeping up the motivation<br>5. Comprehensive discharge planning and information will reduce the confusion in stroke patients	Psychological stress<br>Effective information dissemination creates less stress<br>Patient motivation is essential for rehabilitation<br>Planning reduces stress and confusion<br>Mental health matters
1. Patients often feel vulnerable when they go home from hospital after a stroke<br>2. Thorough diagnosis of TIA's is important in determining a future plan for the patient	Patients are vulnerable<br>Ongoing, quality care<br>Continual care<br>Patient follow up provides a means of reducing future presentations
